# Chemical processes and sustainability of rice-shrimp farming on saline acid sulfate soils in mekong delta

**DOI:** 10.1016/j.heliyon.2023.e13532

**Published:** 2023-02-06

**Authors:** Ngo Phuong Ngoc, Le Van Dang, Nguyen Van Qui, Ngo Ngoc Hung

**Affiliations:** a Department of Plant Physiology-Biochemistry, College of Agriculture, Can Tho University, Can Tho City, Viet Nam; b Department of Soil Science, College of Agriculture, Can Tho University, Can Tho City, Viet Nam

**Keywords:** Na^+^ desorption, Na^+^ hydrolysis, Na-soluble saturated extract, Na^+^ transformation, Soil and water indicators, Sulfurization

## Abstract

In the history of rice-shrimp farming (RSF) in the Mekong Delta, in saline acid sulfate soils (ASS), RSF has proved its sustainability as there is no long-term accumulation of acidity and salinity that adversely affect RSF production. However, the soil processes involved in this phenomenon are not well understood. This study aimed to determine the significant changes in soil and water chemical indicators associated with soil processes during different stages of RSF. Sampling was conducted in six stages to determine the pH and electrical conductivity (EC) of canal and field water, and the pHe, ECe, and sodium-soluble saturated extract (Na-sol) of surface soil (surS) and subsurface soil (subS) in the low-salinity area (LSA) and high salinity area (HSA) of Bac Lieu province. Putative exchange (exch.) Na^+^ hydrolysis occurs at the beginning of the rainy season when excess salts are leached. This drastically increases the pH of the field water by 0.6 pH units, from 8.2 to 8.8 in stages 1 to 2, respectively. Putative sulfurization has been shown to occur in subS in both LSAs and HSAs, with a significant decrease of 0.5 pH units from stages 4 to 5, leading to a decrease in the Na-sol of subS through exch. Na^+^ transformation. Simultaneously, with active soil preparation and liming for RSF, chemical processes such as exch. Na^+^ hydrolysis, sulfurization, and Na^+^ transformation are the main factors promoting exch. Na^+^ desorption, which help to reduce the long-term build-up of salinity and acidity that adversely affect RSF production. Although gypsum is considered an effective material in improving saline soils, in Vietnam, gypsum is the liming material of CaSO_4_; however, it is not commonly used in agriculture because of its high cost. By using CaCO_3_ or dolomite as a liming material in saline ASS, the dissolution of lime by sulfuric acid can provide a good opportunity for soil improvement and rice growth in RSF.

## 1 Introduction

The rice-shrimp farming (RSF) model in the Mekong Delta (MD) is concentrated mainly in the coastal provinces, with an area of more than 220,000 ha. About five provinces have large rice-shrimp areas, of which the largest area is Kien Giang (100,000 ha), followed by Bac Lieu (40,000 ha). Every year, this model can yield around 0.5 tons/ha/year of shrimp and 4.5 tons of rice/ha/crop, with a profit of about 35–50 million VND/ha (1500–2300 USD) [1].

In the coastal MD, the soil of Bac Lieu was developed on the flood plain of the Bassac River in the central area of the MD, which was formed by fluvial deposition 4000–3000 years bp [2,3]. With inundation by brackish water along the coastal line with sufficient organic matter and sediments containing metal cations, and sulfate and pyrite (FeS_2_) being produced [2], Bac Lieu province is under salinity intrusion from the sea, which is overlaid with acid sulfate soils (ASS); therefore, gross processes such as sulfurization and salinization appear in Bac Lieu.

Despite these saline and acidic conditions, RSF started in Bac Lieu province in 2001 and developed rapidly owing to the high returns of shrimp production accompanied by conformity in rice production in the seasonally brackish regions. Some advantages have been recorded in this model, for example, diseases of both shrimp and rice were reduced, profit was increased, and clean production was provided to meet the demands of domestic and international markets. Moreover, after shrimp are harvested, rice cultivation during the rainy season can prolong the life span of land use because the salt has been removed and organic matter from the rice straw remains in the soil [4]. However, in the farming practice, instead of keeping the anaerobic sulfide material under saturated conditions, shrimp ponds need to be prepared for stocking post-larvae; the duration of pond drying is about 30 days (starting 7–10 days before rice is harvested and 15–20 days for pond preparation). Lowering the water table in this soil can lead to intense sulfurization, and by re-flooding after preparation for stocking post-larvae, large amounts of sulfate salts will be formed in the soil [5,6]. Therefore, it is necessary to evaluate the acidity and salinity of the soil.

The dynamics of salinity in rice-shrimp fields depends mainly on the rainfall and salinity of the surrounding canal network. Hydrological conditions in Bac Lieu province are diverse because of a series of sluice gates and seasonal dams constructed since 1994, which aim to regulate saline water intrusion. The salinity dynamics of the brackish water zone are different between the low-salinity area (LSA) and high salinity area (HSA).

The objectives of this study were to (i) identify the significant changes in soil and water chemical indicators associated with soil processes during the different stages of RSF activity; and (ii) evaluate the mechanism that reduces the build-up of salinity and acidity in the soils.

## 2 Materials and methods

### 2.1 Ecological zones

**Fig. 1:**
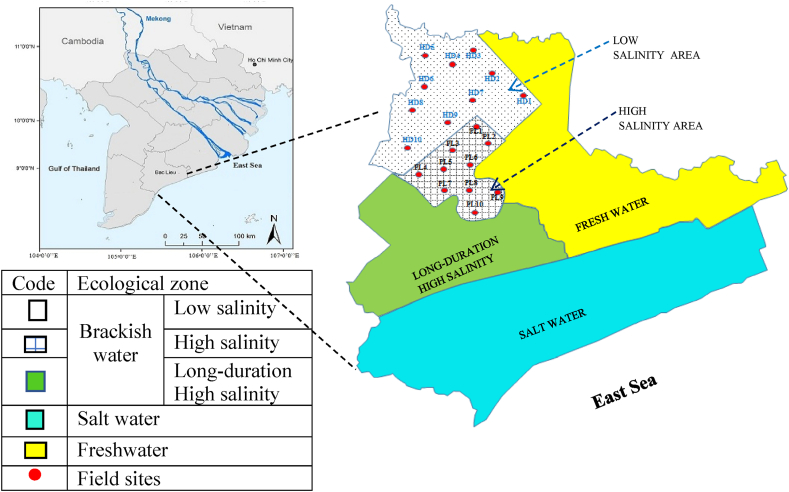
Agro-ecological map of Bac Lieu province. The red points in the LSAs (HD1–HD10) and HSAs (PL1–PL10) indicate the rice fields of this research.

The study area is Bac Lieu province, located in the southern MD (Fig. 1). The land surface is flat (with an elevation of 0.2–0.8 *m* above the mean sea level) and has a dense irrigation canal network. There are numerous seasonal dams and sluices to control saline and freshwater flow. At the East Sea, side sluices have not been built, so this side is still intruded by saline water. However, in the eastern part of the province, the supply of freshwater for rice and upland crops is delivered from the Bassac River through the main canal. In general, the province is divided into three different areas (Fig. 1): (i) the freshwater area in the east; (ii) the brackish area including short-duration salinity (low and high) and long-duration high salinity in the west; and (iii) the saline area in the south [7,8].

In the brackish water zone, the duration of saline water intrusion at low and high salinity areas’ ranges from 3 to 6 months and long-duration high salinity area lasts for over 6 months. However, salinity in the low salinity area (LSAs) is 4‰–6‰, while the high salinity area (HSA) and long-duration high salinity area are more than 10‰ (Table S1).

In the brackish water zone, LSAs and HSAs were chosen for this study. The research sites of LSAs located in Hong dan (HD) district include 10 field sites (HD1 to HD10), and those of HSAs located in Phuoc long (PL) district include 10 field sites (PL1 to PL10). The GPS coordinates in the location description for the HD1–10 and PL1–10 are provided in Table 1.

**Table 1:** GPS descriptions of survey sites in LSAs and HSAs.

No.	Hong Dan (LSA)		Phuoc Long (HSA)	
	Site code	Location	Site code	Location
1	PL1	9°32′17.2″N 105°29′00.3″E	HD1	9°27′31.8″N 105°25′18.6″E
2	PL2	9°32′26.9″N 105°26′15.3″E	HD2	9°26′26.0″N 105°26′27.8″E
3	PL3	9°34′48.3″N 105°24′36.4″E	HD3	9°26′13.8″N 105°23′29.8″E
4	PL4	9°33′41.9″N 105°22′28.5″E	HD4	9°22′51.4″N 105°19′54.7″E
5	PL5	9°33′47.4″N 105°20′08.9″E	HD5	9°23′03.6″N 105°21′36.1″E
6	PL6	9°31′15.0″N 105°20′39.1″E	HD6	9°24′26.5″N 105°24′48.9″E
7	PL7	9°31′06.5″N 105°24′36.4″E	HD7	9°21′06.5″N 105°21′23.7″E
8	PL8	9°29′26.5″N 105°19′25.0″E	HD8	9°21′56.5″N 105°24′01.9″E
9	PL9	9°28′15.8″N 105°22′40.3″E	HD9	9°19′53.3″N 105°24′14.3″E
10	PL10	9°25′37.3″N 105°19′10.1″E	HD10	9°21′52.9″N 105°25′01.3″E

**Fig. 2:**
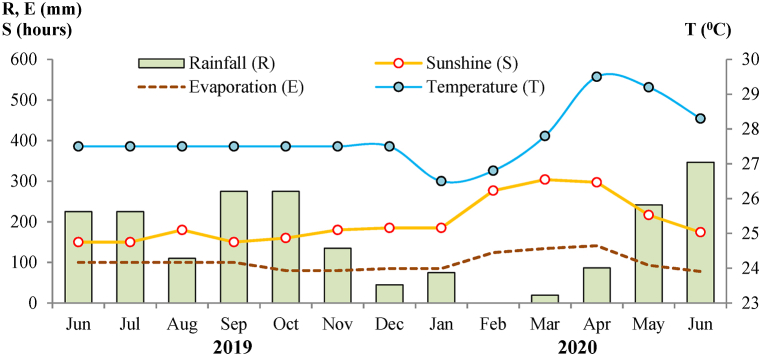
Climatic data of Bac Lieu in 2019–2020 [9].

The study sites are situated in the tropical monsoon area, with two distinct seasons: the rainy season is from May to November and the dry season is from December until April of the following year. The average annual rainfall is 2000–2300 mm. The average temperature is 26 °C, with maximum and minimum temperatures of 30 °C and 26.5 °C, respectively. The yearly sunshine is 2500–2600 h. The average humidity during the dry season is 80%, which increases to 85% during the rainy season [9]. Climatic data of Bac Lieu in 2019–2020 are presented in Fig. 2.

During the dry season, the study sites of rice production face the annual drought combined with saline intrusion from the rivers and canals. Although more than 93% of the total annual rainfall occurs mainly in the rainy season, drought in the MD can occur in the middle of the rainy season, especially in August; the duration could be 7–10 days [10].

### 2.2 The practice of RSF

**Fig. 3:**
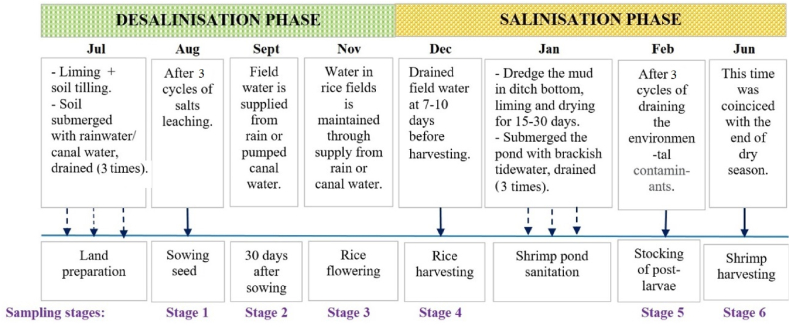
Schematic of stages of RSF practice in LSAs and HSAs, Bac Lieu province.

In practice, every year, the RSF system in LSAs and HSAs in the coastal MD consists of two phases (desalinization and salinization), with six sampling stages (stages 1–6) and two cultivation cycles (rice: July–December; shrimp: January–June) (Fig. 3).

In Bac Lieu, the average cultivation area of the model is 1.0–2.5 ha. A well-known local rice (Oryza sativa) variety, such as “*one red bush*,” and other modern varieties (OM5451, OM2017, HS182) that are salt tolerant are recommended to be grown in this area. The land is prepared after 2–3 rainfalls at the beginning of rainy season. Farmers are recommended to apply CaCO_3_ or dolomite in combination with soil tilling. The lime mostly used in this research area was a commercial product made from limestone [11], with CaO and MgO contents of about 54%–57% and 17%–21%, respectively. The amount of liming material needed is 1.0–1.5 Mg ha^−1^ [12]. During land preparation, the soil is submerged with rainwater or pumped canal water (CW; 2–3 days) to a depth of 10 cm above the field surface. Salt is leached by pumping water from the field to the canal, and the salt leaching cycle is performed three times. Farmers start sowing seed from August to September for long-growth period rice varieties, such as “a red bush” (duration 120 days), which is a local rice variety; or from September to October for short-growth period rice varieties.

Approximately 7–10 days before rice harvesting, water is drained from the field to facilitate harvesting. After rice harvesting, farmers dredge the mud in the ditch bottom. An amount of 0.9–1.3 mg ha^−1^ of CaCO_3_ is cast over its bottom and the pond is dried for 15–30 days. After that, the pond is filled with brackish tidewater, submerged in the soil for 5–7 days, and drained. This procedure is repeated about three times. The purpose of draining before the stocking of shrimp (*Penaeus monodon*) post-larvae is to remove the environmental contaminants that could be harmful to organisms in the soil.

### 2.3 Rice-shrimp model in field construction

**Fig. 4:**
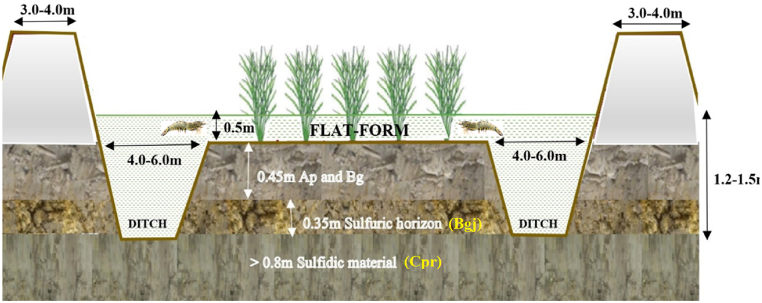
The field transact of the RSF model in ASS in Bac Lieu province.

To maintain water in the pond, an embankment was built and the surrounding canal area was expanded (3.0–4.0 *m*), with the water depth being at least 1.2 *m* in the ditch and 0.5 *m* in the central platform (Fig. 4). The platform-to-ditch ratio was about 80% [12]. The construction of ASS at both LSAs and HSAs was identical, where an underground water table was observed at a depth of approximately 80 cm in the profile. However, January of every year is the time for pond drying to stock post-larvae; therefore, the water table depths of the soils were deeper than 80 cm from the top soil layer.

The soil profile description in the LSA zone has a morphology identical to that of the HSA, for example, the top soil was composed of Ap and Bg layers with depths of 0.20 and 0.25 *m*, respectively. Sulfuric horizon with yellow (2·5 Y 8/8) Jarosite mottles infillings in the soil matrix and root holes above the sulfide layer, the depth of these sulfuric layer (named as Bgj horizon) were found at 0.45–0.8 *m* below the soil surface. The underlying potentially ASS layer is gray (GLEY1 6/10 y) silty clay with some organic materials (7.5Y 3/3); sulfidic material was identified by H_2_O_2_ (pH less than 2.0), which appeared at greater than 0.8 *m* below the soil surface (named as the Cpr horizon). The soil type examined was Typic Sulfaquepts [13,[14]a], [14]b]].

### 2.4 Water and soil sampling

The field monitoring was conducted from August 2019 to June 2020. The selected sites of HD (n = 10) and PL (n = 10) are presented in Fig. 1. Soil and water sampling was conducted in six stages: (i) sowing rice seed; (ii) 30 days after sowing; (iii) rice flowering; (iv) rice harvesting; (v) stocking of post-larvae; and (vi) shrimp harvesting. The six sampling stages are presented in Fig. 3.

#### 2.4.1 Water sampling

Field water (FW) and CW were sampled in the morning (6–8 am), just 1 day before sowing rice seed (stage 1). Water samples of each field surface or each canal collected from five sites were mixed together in a plastic bottle. The pH and electrical conductivity (EC) of the water in the bottle were determined directly in the field. Hanna digital meters (HI 991301 Model) were used to measure the water pH and EC of the water samples. A total of 240 water samples, including FW and CW, were collected (2 areas × 10 fields/area × 6 sampling stages × 2 types of water samples).

#### 2.4.2 Soil sampling

To determine the dynamics of soil ECe, pHe, and sodium-soluble saturated extract (Na-sol) from LSAs and HSAs at each field, soil was taken at five locations diagonally and composited into one sample weighing about 2.0 kg. Plastic bags were used to store fresh soil samples that were then transported to the laboratory within 1–2 days. Six stages of soil sampling were performed from two saline areas (n = 10 for each area). Soil depth was sampled at the surface (surS; 0–20 cm) and subsurface layers (subS; 20–45 cm). The total number of soil samples was 240 (2 areas × 10 fields/area × 6 sampling stages × 2 soil depths).

To determine the physical and chemical properties of soil from LSAs and HSAs, soil was sampled at the two salinity areas (n = 10 for each area) (Fig. 1). Soil was collected in layers of depth 0–20 and 20–45 cm. The total number of soil samples was 40 (2 areas × 10 fields/area × 2 soil depths).

To determine the chemical characteristics of soil profiles from LSAs and HSAs, at the onset of the rainy season in 2019, before performing soil profile descriptions and soil physicochemical analyses for the two soil areas, an exploratory survey was first carried out. Based on the multiple observation points to probe, the most representative soil profile for the soil area was chosen [15]. From 10 drill bits probed in LSAs (HD1–HD10), HD7 was chosen. Similarly, from 10 drill bits probed in HSAs (PL1–PL10), PL7 was chosen. Soil sampling in each soil profile was performed in four soil horizons. The total number of soil samples was 8 (1 profile/area × 2 areas × 4 soil horizons).

To study the dynamics of soil exchangeable cations (K^+^, Na^+^, Ca^2+^, and Mg^2+^) in HSAs, three sites were selected (PL7, PL8, and PL9, as indicated in Fig. 1). The soil depth was 0–20 and 20–45 cm. Six stages of soil sampling (Fig. 3) were performed. The total number of soil samples was 36 (3 sites × 6 sampling stages × 2 soil depths).

The soil samples were placed in sealed plastic bags and the samples were dried in a fan-forced oven at 80 °C for a few days until a constant mass was achieved. Dried soil samples were sieved (<2 mm) [16].

### 2.5 Soil physical and chemical analyses

The soluble and readily dissolvable salts in water or soil solution significantly influence EC (in S m^−1^). The most relevant measurement of soil salinity to plant growth is the EC of a saturated paste extract (ECe), which is a measure of salt concentration in the soil solution (mg L^−1^) [17]. In the laboratory, just enough deionized water was added to the soil sample to achieve saturation. After more than 4 h for equilibration, a vacuum pump was used to extract water in the soil (saturated extraction) [18]. The extracts were measured using a pH meter (Metrohm 744) and an EC meter (Horiba B-173); the resultant values are the pHe and ECe (S m^−1^), respectively. The Na-sol was extracted with a saturated paste extract and determined by atomic absorption spectrophotometry (iCE 3000 Series).

For the measurement of putative exchange (exch.) cations in saline soils, the method was based on the difference between the soluble and extractable cations [19]. To have soluble Na, K, and Ca, soil was extracted with deionized water at a ratio of 1:10 (soil:water) and shaking for 1 h at 120 rpm. Then, the soil solution was centrifuged at 8000×*g* and passed through a filter paper (Advantec No. 5C). To have extractable cations, 2.5 g soil sample was extracted three times with 0.1 M BaCl_2_ solution (each time with 30 mL and shaking for 1 h at 120 rpm). The ions in the soluble and extractable cations were determined by atomic absorption spectrophotometry. The soil exch. Cations were then calculated as the difference in concentration between total extractable and soil solution cations. The compulsive exchange method was used to measure CEC [20].

The Robinson pipette method was used for soil particle size analysis. After the soil sample was air dried and sieved at 2 mm, based on Stokes’ law, the different particle sizes were collected, and the sand (0.05–2 mm), silt (0.002–0.05 mm), and clay (<0.002 mm) fractions of the soil sample were determined. Saturated hydraulic conductivity (K_sat_) was measured by applying the constant-head cylinder method in undisturbed soil samples, which were saturated prior to determination. The determination of soil organic carbon (OC) was based on the wet oxidation of chromic acid [21].

### 2.6 Data analyses

Excel was used to perform the *t*-test, standard deviation (SD), standard error (SE), and regression analysis in this study. Regression analysis was used to predict K_sat_. The *t-*test was run on the data to reveal any statistically significant differences (p ≤ 0.05) in the basic characteristics of LSAs and HSAs. The average of replicates (*n* = 10) is presented in Table 2, and the average of replicates (*n* = 10) is presented with SE in all figures in soil and water dynamics, except in Fig. 10, Fig. 11, where the replicates are three.

**Table 2:** Some physical and chemical properties of the surS and subS (Stage 1) in Hong Dan (LSA) and Phuoc Long (HSA) study sites. Each soil sample includes surS (n = 10) and subS (n = 10) from Bac Lieu, August 2019.

Sites	Hong Dan (LSA)							Phuoc Long (HSA)					T-test^#^
Depth	Item	Unit	Mean	Min	Max	SD	CV (%)	Mean	Min	Max	SD	CV (%)	
surS (0–20 cm)	Sand	%	2.3	1	3	0.67	29.3	7.6	2	11	2.78	36.5	**
	Silt	%	45.1	38	55	5.2	11.5	37.5	30	52	6.15	16.3	*
	Clay	%	52.6	43	60	5.17	9.8	54.9	39	65	6.82	12.4	ns
	K_sat_	mm h^−1^	42.6	27.3	60.6	11.1	26.1	41.3	8.04	75.8	19.5	47.1	ns
	OC	%	2.39	1.68	3.79	0.64	26.7	2.57	1.63	3.59	0.51	19.7	ns
	CEC	cmolc^+^ kg^−1^	18.7	13.2	22.7	2.5	13.3	22.8	14.1	24.2	2.66	11.7	**
subS (20–45 cm)	Sand	%	3.2	2	4	0.79	24.6	6.5	3	9	2.55	39.2	**
	Silt	%	38.3	35	47	3.4	8.8	34.2	26	43	5.63	16.4	ns
	Clay	%	58.5	50	63	3.5	5.9	59.3	54	65	3.59	6	ns
	K_sat_	mm h^−1^	16.2	8.04	35.4	7.59	46.9	15.3	4.22	25.2	6.41	42	ns
	OC	%	2.35	1.75	2.64	0.26	11.2	2.41	1.78	3.03	0.45	18.7	ns
	CEC	cmolc^+^ kg^−1^	19.1	14.1	21.6	2.25	11.7	21.9	18.4	24.3	2.25	10.2	*

Ns: non-significant; ^#^significant at *0.05; **0.01.

## 3 Results

### 3.1 Soil physical and chemical characteristics

In this study, multiple regression analysis was used to predict K_sat_ by considering the clay content and OC content as independent variables. Both the variables were significant predictors for K_sat_; the model is presented in Eq. (1) as follows:

(1) $${Y\;(K}_{\text{sat}})=\begin{array}{cccc} -2.84\text{Clay}\left(**\right) & +8.28\text{OC}\left(*\right) & +168.67\left(**\right) & R^{2}=0.76** \end{array}$$

Significant at *0.05 and **0.01,

Where clay is the clay content (%) and OC is organic carbon content (%). In Eq. (1), clay content is the factor causing the decrease in the Ksat value, whereas OC causes an increase in the Ksat value.

The clay percentages in the subS of the LSAs and HSAs (58.5 and 59.3, respectively) were higher than those of the surS (52.6 and 54.9, respectively). On average, the subS of both area soils contained a higher clay content than the surS, and the average clay content was greater than 5% (Table 2). However, the clay contents in surS and subS of both LSAs and HSAs were not significantly different. A higher clay content led to lower K_sat_ values in subS of LSAs and HSAs. There was a difference in K_sat_ values between subS and surS of the two area soils. The K_sat_ of subS in the LSAs and HSAs (16.2 and 15.3 mm h^−1^, respectively) were lower than those of surS in the LSAs and HSAs (42.6 and 41.3 mm h^−1^, respectively) (Table 2). The dataset on properties of the soil are presented in Table S2.

Representative soil profiles selected for each land area were constructed according to Ref. [15]. HD7 was chosen from 10 drill bits probed in LSAs (HD1–HD10). Similarly, PL7 was chosen from 10 drill bits probed in HSAs (PL1–PL10). Table 3 presents the chemical properties of soil profiles from LSAs and HSAs. In both soil profiles, the pHe values of Ap and Bg horizons were greater than 5, whereas the pHe values decreased to below 3.5 when extended into the sulfuric zones. The pHe value of Bgj in LSA soil was 2.91 and that in HSA soil was 3.15. The pHe value of Crp in LSA was 3.29 and that of HSA was 3.77. At 2–3 days before sowing seed, the ECe values of surS in LSA and HSA were 0.6 and 1.7 S m^−1^, respectively, and the exchangeable sodium percentage (ESP) values of surS in LSA and HSA were 12% and 31%, respectively (Table 3).

**Table 3:** Chemical properties of LSA and HSA soil profiles during stage 1 of RSF.

Site	Horizon	Depth	pHe	ECe	CEC (cmolc^+^ kg^−1^)	Exchangeable cation				ESP (%)
						Na^+^	K^+^	Ca^2+^	Mg^2+^	
		(cm)		(S m^−1^)		(--------cmols^+^ kg^−1^-------)				
HD7 (LSA)	Ap	0–20	6.86	5.55	19.5	2.34	0.58	3.64	11.1	12
	Bg	20–45	6.54	10.1	20.1	2.85	0.74	3.71	12.2	14
	Bgj	45–80	2.91	7.88	16.3	2.44	0.47	5.91	2.88	15
	Crp	>80	3.29	7.95	15.8	2.9	0.83	3.02	7.96	18
PL7 (HSA)	Ap	0–20	5.90	17.6	24.2	7.6	1.15	3.78	5.64	31
	Bg	20–45	5.07	8.11	24.2	6.55	0.77	4.15	5.16	27
	Bgj	45–80	3.15	4.78	21.4	12.7	0.74	2.64	7.68	59
	Crp	>80	3.77	5.48	22.6	7.36	0.89	4.58	9.74	33

The pHe, ECe, and ESP of Ap horizon in the LSA were 0.7, 0.6 S m^−1^, and 12%, respectively, and those in the HSA were 0.6, 1.8 S m^−1^, and 31%, respectively (Table 3). Therefore, the LSA and HSA soils could be classified as saline soil and saline–sodic soil, respectively. The pHe values of the Bgj horizon in the LSA and HSA soil profiles were 2.91 and 3.15, respectively; both soils were classified as Typic Sulfaquepts.

### 3.2 Relationship between CW EC and FW EC

The salinity in the coastal MD has two phases of the salinity cycle: the “desalinization phase” that starts from the onset of the rainy season (May) and finishes at the end of the rainy season (November); and the “salinization phase” that starts with the recession of the rain (December) and finishes when the salinity reaches the highest at the end of the dry season (April) (Fig. 2). In the desalinization and salinization phases, the EC level of FW varied along with that of CW.

**Fig. 5:**
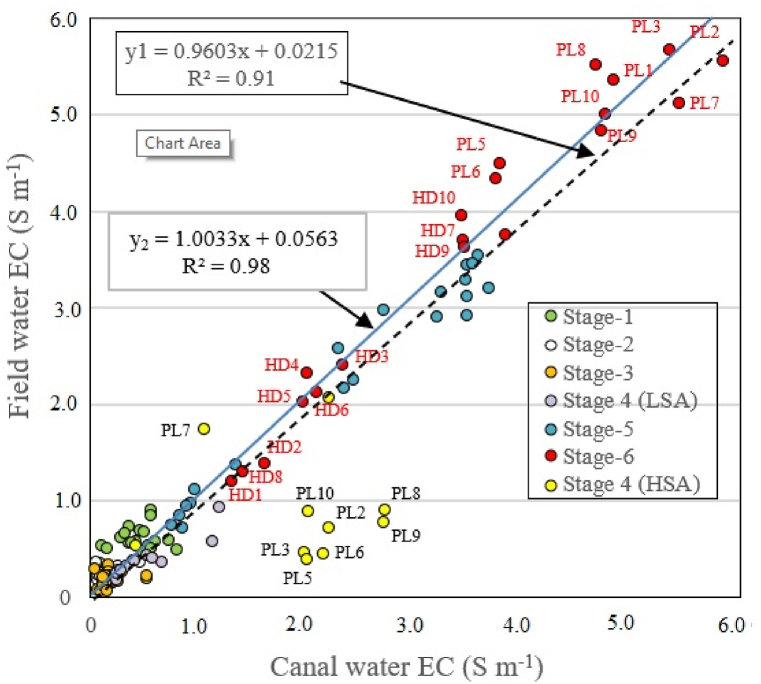
Correlation between the EC of FW and that of CW during six stages of farming. Y_1_ includes the whole data set from the two areas; y_2_ excludes stage 4 data from the HSA (n = 10 for each data point).

**Fig. 6:**
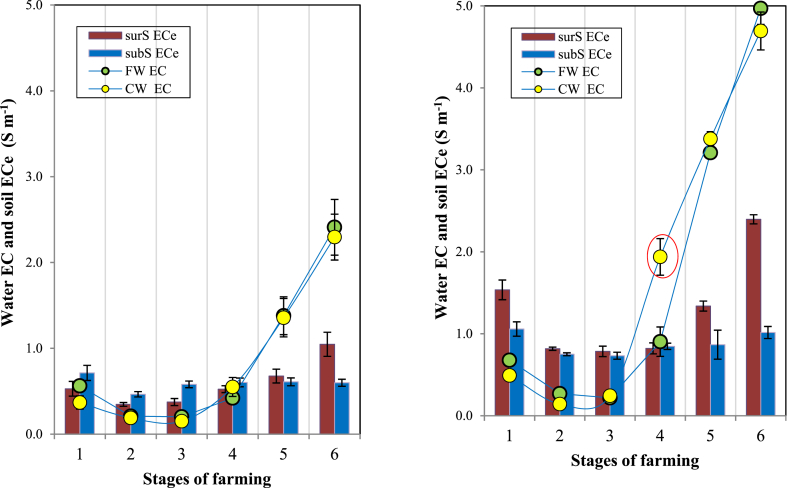
Dynamics of water EC (CW, FW) and soil ECe (surS, subS) in (a) LSAs and (b) HSAs in Bac Lieu, 2019. The vertical bar indicates the mean ± SE of each treatment (n = 10) at p ≤ 0.05. The red circle in Fig. 6b shows a specific slow increase in FW EC, whereas CW EC increases continuously.

During stages 2 and 3 (30 days after sowing and rice flowering, respectively), the flushing of saline water from FW by precipitation was favorable for rice growth; the EC of FW and CW in the two stages was less than 5 S m^−1^ (Fig. 5, Fig. 6). In stages 5 and 6 (supply of post-larvae and shrimp harvesting, respectively), surrounding CW was taken to the field for shrimp cultivation; the EC of both FW and CW in stage 6 could reach as high as nearly 6.0 S m^−1^.

A correlation analysis between FW and CW was performed. When the whole data set from the two areas was included, the results yielded a good match (R^2^ = 0.91) with the straight linear equation *y*_*1*_ = *0.9603x* + *0.2148* and a better match (R^2^ = 0.98) with the straight linear equation *y*_*2*_ = *1.0033x* + *0.5626*.

### 3.3 Dynamics of water EC and soil ECe

During the flushing period, the salinity from the field was removed mainly by rainwater; therefore; the EC in FW became as low as the EC in CW (Fig. 6a–b). In LSAs, the EC values of FW in stages 1, 2, and 3 decreased steadily from 0.6 to 0.2 to 0.2 S m^−1^, respectively, and those of FW in HSAs also decreased gradually from 0.7 to 0.2 to 0.2 S m^−1^ in stages 1, 2, and 3, respectively (Fig. 6a).

In stages 1, 2, and 3, the ECe of surS in LSAs is always lower than that of subS, but in HSAs, it is the opposite. In LSAs, in stages 1, 2, and 3, the ECe values of surS were 0.5, 0.4, and 0.4 S m^−1^, respectively, where were lower than those of subS in stages 1, 2, and 3, at 0.7, 0.5, and 0.6 S m^−1^, respectively (Fig. 6a). On the contrary, for HSAs, in stages 1, 2, and 3, ECe values of surS were 1.5, 0.8, and 0.8 S m^−1^, respectively, which were higher than those of subS in stages 1, 2, and 3, at 1.1, 0.8, and 0.7 S m^−1^, respectively (Fig. 6b).

The lowest soil ECe of the two areas was recorded in stage 2 (30 days after sowing) and stage 3 (rice flowering); in LSAs, the ECe values of surS were less than 0.4 S m^−1^, whereas in HSAs, the ECe values of surS were in the 0.7–0.8 S m^−1^ range.

### 3.4 Relationship between water pH and Na-sol

**Fig. 7:**
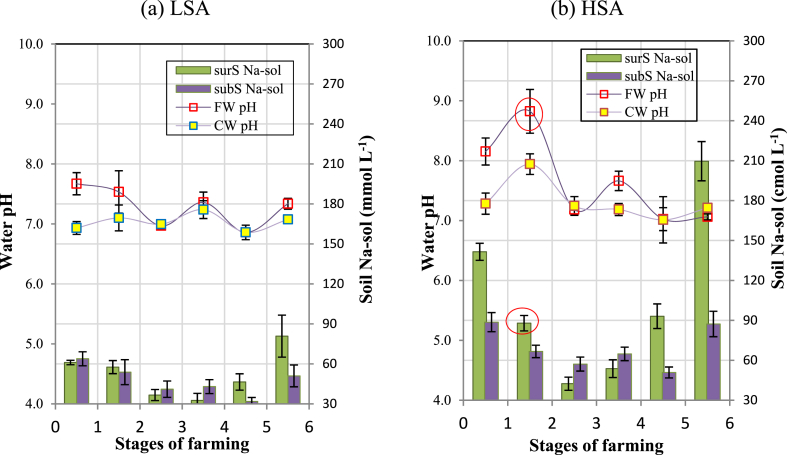
Dynamics of water pH (CW, FW) and soil Na-sol (surS, subS) at (a) LSAs and (b) HSAs in Bac Lieu, 2019. The vertical bar indicates the SE ± of each treatment (n = 10) at p ≤ 0.05. Bac Lieu, 2019. The red circle in Fig. 7b shows a specific decrease in surS Na-sol, whereas the FW pH increased.

In general, during the six stages of RSF, the pH of FW in both LSAs and HSAs was below 8.0, except in stage 2; the degree of variation in pH in the two regions is different. In HSAs, the pH of FW increased spectacularly from stages 1 to 2, from 8.2 to 8.8; whereas in LSAs, the pH increase from stages 1 to 2 was only 7.3 to 8.0 (Fig. 7a–b). After stage 2, the pH of both FW and CW decreased steadily because FW was flushed continuously during the rainy season. The pH of CW showed the same trend as that of FW but had a lower parallel value. The variation trend of CW pH in six stages of the two areas was the same as that of FW because the direct exchange was often between FW and the surrounding CW.

**Fig. 8:**
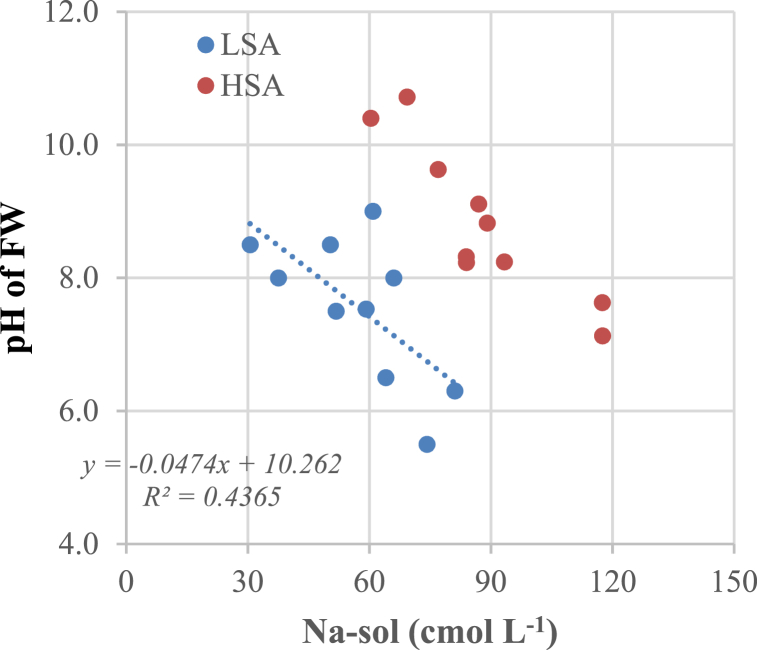
Correlation between Na-sol and pH of FW in LSA and HSA soil.

The fluctuations of pH and Na-sol in LSAs are less obvious than those in HSAs (Fig. 7a and b). In HSAs, there was a continuous decrease in stages 1, 2, and 3 in the Na-sol of surS, at 142, 88, and 42 mmol L^−1^, respectively. Fig. 8 shows that there is a negative regression correlation between Na-sol and the pH of FW, because Na-sol of LSAs is lower than that of HSAs; therefore, the two soils have two different linear correlations. The equation of LSAs is y = −0.0474x + 10.262, with the regression coefficient (R^2^ = 0.4365) being rather low; whereas the equation of HSAs is y = −0.0566x + 13.798, with the regression coefficient (R^2^ = 0.8078) being a better fit.

### 3.5 Relationship between soil pHe and Na-sol

**Fig. 9:**
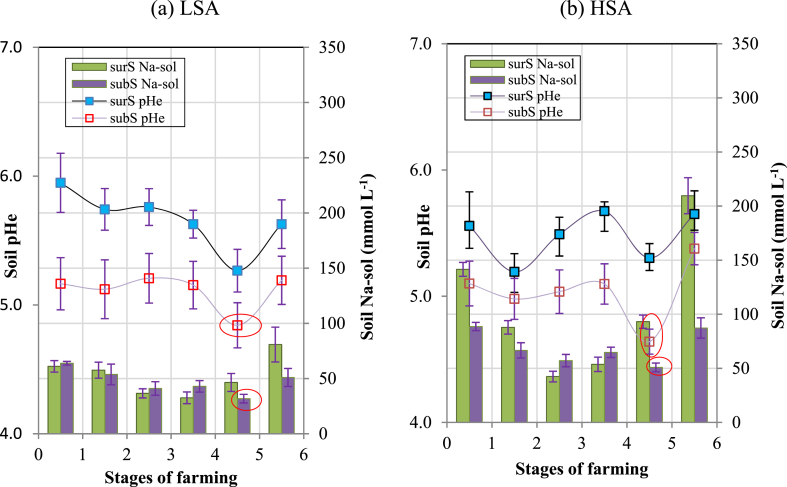
Dynamics of soil pHe and soil Na-sol in surS and subS in (a) LSAs and (b) HSAs in Bac Lieu, 2019. The vertical bar indicates the mean ± SE of each treatment (n = 10) at p ≤ 0.05. The red circles in Fig. 9a–b shows the specific decrease of subS Na-sol that occurs along with the decrease of subS pHe.

In stages 1 to 4, the soil pHe of surS and subS in LSAs and HSAs are low and appeared in the range of 5.0–6.0 (Fig. 9a–b). However, in stage 5, the soil pHe of surS and subS in LSAs and HSAs declined drastically; the pHe of subS tended to be lower than that of surS. In LSAs, the pHe values of subS and surS were 4.9 and 5.3, respectively, and in HSAs, these values were 4.7 and 5.3, respectively (Fig. 9a–b). In general, in stage 5 of both saline areas, the average pHe of subS was 0.5 pH units lower than that of surS. After stage 5, the pHe of soils in the two areas increased back to their original value.

**Fig. 10:**
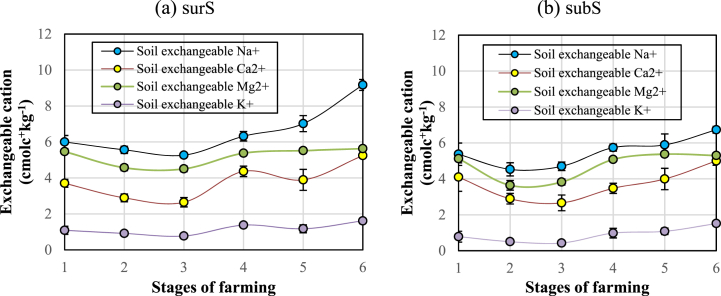
Dynamics of soil exch. Na^+^, K^+^, Ca^2+^, and Mg^2+^ in (a) surS and (b) subS of HSAs. The vertical bar indicates the mean ± SE of each treatment (n = 3) at p ≤ 0.05.

**Fig. 11:**
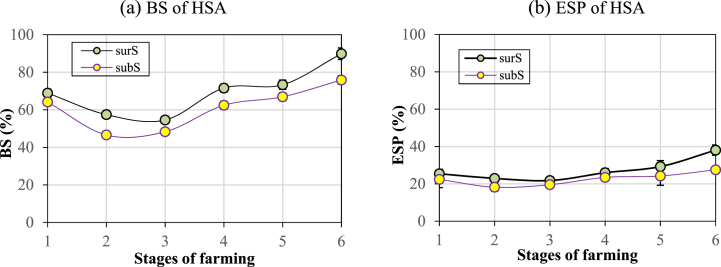
Dynamics of (a) BS and (b) ESP in surS and subS of HSAs. The vertical bar indicates the SE ± of each treatment (n = 3) at p ≤ 0.05.

It was noted that the Na-sol of the two salinity areas varied along the soil pHe until stage 4 (Fig. 9a–b). However, in LSAs at stage 5, the Na-sol of subS dropped from 43 mmol L^−1^ (stage 4) to 31 mmol L^−1^ (stage 5) (Fig. 9a), and in HSAs, the Na-sol declined from 65 mmol L^−1^ (stage 4) to 51 mmol L^−1^ (stage 5) (Fig. 9b). The decrease in Na-sol in stage 5 for LSAs was related to the decrease of pHe (Fig. 9A), which can be seen more clearly than for HSAs (Fig. 9b). It was assumed that the Na-sol in subS was partly removed.

### 3.6 Dynamics of soil exch. Cations in HSA soils

In stages 1 and 2, there was a slight decline in the basic cations in the surS and subS (Fig. 10a and b). From stages 3 to 4, the exch. Ca^2+^ and Mg^2+^ in surS and subS continued to increase, then their contents were relatively stable, in the range of 4–5 mmol kg^−1^. The contents of exch. Na^+^ in the subS in stages 4, 5, and 6 increased slightly, at 5.74, 5.89, and 6.73 mmol kg^−1^, respectively (Fig. 10b).

In this study, multiple regression analysis was used to predict exch. Na^+^ by considering the Na-sol and soil pHe as independent variables. Both these variables were significant predictors for Na^+^; the model is presented in Eq. (2) as follows:

(2) $${Y\;(\text{Exch}.\;\text{Na}}^{+})=+0.016\text{Na}-\text{sol}+0.504\text{pHe}+2.129\left(*\right)\left(*\right)\left(***\right)R2=0.75***$$

Significant at 0“***,” 0.001“**,” and 0.01“*”

Where Na-sol and pHe are the soil values extracted from the saturated paste. In Eq. (2), the Na-sol concentration and pHe are the factors that cause the increase in the exch. Na+.

### 3.7 Dynamics of base saturation (BS) and ESP in HSA soils

The BS of surS decreased rapidly during the rainy season, to 69%, 57%, and 55% for stages 1, 2, and 3, respectively. Then, when salt water intruded, BS started to increase continuously from stages 4 to 5 and 6 by 60%, 80%, and 95% respectively (Fig. 11a). The BS of subS had a parallel development with the BS of surS and was always about 10% lower.

The ESP of surS was high in stage 1 (25.4%) and decreased gradually in stages 2 (23%) and 3 (22%) (Fig. 11b); then, it started to increase in stage 4 and reached up to 38% in stage 6. The ESP of subS in all the six stages had a parallel development with the ESP of surS and was always lower than that of surS.

## 4 Discussion

### 4.1 Soil physical and chemical characteristics

K_sat_ is a critical variable affecting the hydrology and acid export dynamics of drained ASS [22]. In near surface of ASS, although *K*_*sat*_ values can be estimated from correlated soil properties, such as texture, bulk density, organic matter, particle size distribution, and structure descriptions [23], the pore size distribution and connectivity of macropores can affect solute flux pathways; therefore, the main transport pathway of acidic soluble materials to drainage systems in soils with macropores in the sulfuric horizon is by saturated water flow [24,25].

According to Ref. [12], the K_sat_ of surS was classified as “high” and that of subS was classified as “moderately high.” The results of this study found that the CV (%) values of K_sat_ in surS and subS (26.1 and 46.9, respectively) in LSAs were as high as those of K_sat_ in surS and subS (47.1 and 42.0, respectively) in HSAs. As indicated by Ref. [22], *K*_sat_ in sulfuric horizons can be very high and is variable within individual coastal floodplains.

In saline-affected soils, the classification and assessment of the effects of soil salinity on crops are usually based on the ECe. Saline soil is created with salt content derived from sea water; the salt composition includes four cations (Na^+^, Mg^2+^, Ca^2+^, K^+^) and two anions (Cl^−^ and SO_4_^2−^). These components account for more than 99% of the ions in sea water [26]. The soils are considered saline when the ECe value is above 0.4 S m^−1^, pH is below 8.5, ESP is less than 15 or the sodium adsorption ratio (SAR) is less than 13 in the saturation extract, and usually contain sufficient soluble salts, which adversely affect the growth of most crops if the ECe is above 1.0 S m^−1^ [27].

Traditionally, salt-affected soils were classified into three types: saline, sodic, and saline–sodic. Saline–sodic soil has an ECe value of 0.4 S m^−1^ or greater, a pH value usually lower than 8.5, and ESP greater than 15 [28]. CaSO_4_ requirement can determine the amount of CaSO_4_ to be applied, to ameliorate a sodium problem by using ESP [29]. Furthermore, the SAR can be converted to ESP.

As the high ratio of Na adsorbed on soil colloids is considered to promote its dispersion, thus negatively affecting soil physical properties [30], HSA soil was classified as saline–sodic soil with ESP greater than 15 (Table 2), because Na is a monovalent cation with a large hydrated radius.

With jarosite mottles of a particular color and active ASS mostly having sulfuric horizons at or near their surfaces, the present sulfuric horizon definition requires a pH less than 3.5 [12]. As both soils with the sulfuric horizon occur within a depth of 0–50 cm (Table 3), both soils were classified as Typic Sulfaquepts.

The very low pHe of those Bgj horizons were generated by the hydrolysis of relatively insoluble Fe/Al hydroxy sulfate minerals of jarosite [31]. This acidity is not readily available but is considered to be released slowly by the hydrolysis of jarosite [32,33]. The reaction takes place (Eq. (3)) as follows:

(3) $${\text{KFe}}_{3}{\left(\text{OH}\right)}_{6}{\left({\text{SO}}_{4}\right)}_{2}+3H_{2}O\to 3\text{Fe}{\left(\text{OH}\right)}_{3}+2{\text{SO}}_{4}^{2-}+3H^{+}+K^{+}$$

The pHe values of Crp horizon in the soil profiles of LSAs and HSAs were 3.29 and 3.77, respectively. When a sample of Crp soil horizon is exposed to air owing to drying of the sample for soil analysis, acidity can be produced by the pyrite oxidation reaction (Eq. (4)) as follows [34]:

(4) $${\text{FeS}}_{2}+15/4\;O_{2}+7/2H_{2}O\to \text{Fe}{\left(\text{OH}\right)}_{3}+2{\text{SO}}_{4}^{2-}+4H^{+}$$

In the field survey, hydrogen peroxide could be used to test sulfidic material in fresh soil samples; the Crp horizon in both soils was found by testing the soil sample's Crp with H_2_O_2_; then, the soil reached a pH less than 2.0. As suggested by Refs. [35,36], a simple field test on sulfide materials is as follows: after applying 30% hydrogen peroxide to the fresh sample, within a couple of minutes, it will froth and its pH will fall from neutral to less than 2.5. Although in general, pyrite oxidation generated acidity and proceeded faster in mineral samples [37], note that the hydrogen peroxide test was not effective for soils with high organic matter content because acidity may have been generated by the oxidation of organic matter to organic acids [35,37], which leads to lower pH values.

In general, in the two soils, the clay content in subS was higher than that in surS, resulting in subS having a lower K_sat_ value. Although the pH values of the Ap and Bg horizons in both soil profiles were greater than 5, the pHe value of the diagnostic sulfuric horizon was less than 3.5. According to the US Department of Agriculture, the soil is classified as typical Sulfaquepts. Based on the pHe, ECe, and ESP values of the A horizon, LSA and HSA soils can be classified as saline soils and saline–sodic soils, respectively.

### 4.2 Dynamics of water EC and soil ECe

When the whole data set was included from the two areas, and because limited saline water from the surrounding canal was allowed to enter the rice field, the result was that the EC of FW was lower than that of CW, leading to a weak correlation between FW and CW in stage 4 (Fig. 5). Data points of stage 4 (harvesting stage) were scattered (Fig. 5), with the correlation coefficients (R^2^ = 0.91) not being as high as that when stage 4 data from the HSA were excluded (R^2^ = 0.98).

The “desalinization phase” is characterized by a steady decrease in soil ECe because at the beginning of the rainy season, farmers use rainfall as well as fresh water from rivers to flush residual salinity in the soil. Therefore, the soil ECe of LSAs and HSAs in the “desalinization phase” at the beginning of the rainy season was low; the ECe of land preparation soil (stage 1) was much lower than that of shrimp harvesting soil (stage 6) (Fig. 6a–b). In all six stages, the soil ECe in the HSA was almost twice as high as that of the LSA. In general, the EC of FW go along with CW and EC of CW in LSA (Fig. 6a) was always lower than that of HSA (Fig. 6b). This is because LSAs have freshwater flow from the Mekong River, which is sufficient to displace the saline intrusion from the eastern Bac Lieu province; however, HSAs do not have freshwater flow [38].

In the LSA, in stages 1, 2, and 3, the lower ECe of surS than that of subS (Fig. 6a) was attributed to the leaching/flushing processes, in which the salt was removed more effectively than that of subS. On the contrary, in the HSA, in stages 1, 2, and 3, the ECe values of surS (Fig. 6b) were significantly higher than those of subS. These higher ECe values were attributed to more salts being absorbed into the clay particles and because early rainfall was not sufficient to leach out the salts.

The K_sat_ indicator can help explain the flushing process to remove salt in the soil. As shown in Table 2, in LSAs, the K_sat_ of surS (42.6 mm h^−1^) is higher than that of subS (16.2 mm h^−1^), so the salt in surS is removed more efficiently. Therefore, the ECe values of surS in stages 2, 3, and 4 (0.5, 0.4, and 0.4 S m^−1^, respectively) were significantly lower than those of subS (0.7, 0.5, and 0.6 S m^−1^, respectively) (Fig. 6a). However, the use of the “K_sat_ indicator” is still unclear in explaining that there is no difference in salt content between surS and subS in HSA, evn though the K_sat_ of surS (41.3 mm h^−1^) is higher than that of subS (15.3 mm h^−1^). Therefore, as shown in Fig. 6, the ECe values of surS at stages 2, 3, and 4 were not significantly lower than those of subS.

The accumulation of salts in the root zone can reduce crop growth and yields, and in severe cases, may cause crop failure. Moreover, the high salt composition in soil solution may cause the change in cation composition on the exchange complex of clay particles, which influences soil permeability and tilth [28]. Fig. 6a–b shows that during stages 2 and 3, the ECe of surS in LSAs and HSAs was between 0.7 and 0.8 S m^−1^; however, the EC of FW of those areas in stages 2 and 3 was around 0.2 S m^−1^, which indicates that at this salinity threshold, rice growth is less damaged. As indicated by Ref. [39], at floodwater EC levels >0.2 S m^−1^, rice yield may lose up to 1 ton ha^−1^ per 0.1 unit EC (S m^−1^) for susceptible cultivars, and up to 0.6 ton ha^−1^ per unit EC for tolerant cultivars. Moreover, soils containing sufficient soluble salts (ECe ≥0.4 S m^−1^) may interfere with the growth of most crop plants [19].

The water EC in the “salinization phase” was characterized at the recession of the rainy season by the sharp increase in the EC in CW and FW. In LSAs, in stages 4, 5, and 6, the EC values of FW were not different from those of CW (Fig. 6a). However, in HSAs, in stage 4, the EC value of FW (0.9 S m^−1^) was half that of CW (1.9 S m^−1^) (Fig. 6b), suggesting that since December 12–15, 2019, saltwater intruded into estuaries of the Mekong River with the salinity limit of 4 g L^−1^, approaching as far as 57 km inland [40]. The soil ECe of LSAs and HSAs in the “salinization phase” corresponded to the EC variations of CW and FW; therefore, during the rice harvesting stage, the farmer minimized bringing brackish water into the rice field.

The highest soil ECe of LSAs and HSAs occurred in stage 6 (shrimp harvesting); this time, it coincided with the end of the dry season. From the salts remaining from stage 6 and then in the early stage 1, the ECe of surS in LSAs and HSAs attained values of 0.5 and 1.5 S m^−1^, respectively. A conservative threshold salinity level then for rice transplanting is around 1.0 S m^−1^ [[14]a], [14]b]].

In general, the EC of FW go along with CW; over all the six stages, the soil ECe in HSA soil was almost twice as high as that of LSA soil. The highest soil ECe of LSAs and HSAs occurred at the end of the dry season. As a result, in the early stage 1 in LSAs and HSAs, the salt content remaining in surS is still high; therefore, rainfall and fresh water were used to flush the residual salinity in the soil. In LSAs, the removed salt in surS can be effectively explained by the K_sat_ indicator. Although in stages 2 and 3, the ECe of surS in LSAs and HSAs was in the range of 0.7–0.8 S m^−1^, the EC of FW was about 0.2 S m^−1^, which helped rice to grow with less damage.

### 4.3 Relationship between water pH and Na-sol

In HSAs, there was a sudden increase in water pH of FW in stage 2 (Fig. 7b). As hydrolysis of the exch. Na^+^ takes place when excess soluble salts are leached from the top soil by the rainwater while exch. sodium in the soil still remains, hydrolysis of the exch. Na^+^ leads to an increase in the pH of FW by the alkaline reaction of the soils; this phenomenon reacts similarly to sodic soil. The reaction takes place as follows [41].

$${Soil\;colloid}_{Na}^{Na}+H_{2}O\;\Leftrightarrow {Soil\;colloid}_{Na}^{Na}+{Na}^{+}+{OH}^{-}$$

In this reaction, H^+^ is inactivated by exchange adsorption in soil colloids. The displaced Na does not inactivate OH^−^ ions, which results in increased soil pH. Ca^2+^ and Mg^2+^ ions are more absorbed by soil colloids than Na^+^, but hydrolysis of exch. Ca^2+^ and Mg^2+^ ions is limited in saline soils. However, exch. Na ^+^ are hydrolyzed to a greater extent and produce a higher pH than do exch. Ca^2+^ or Mg^2+^. The hydrolysis of compounds such as CaCO_3_ and MgCO_3_ is limited because of their low solubilities; therefore, the pH in soils is no higher than about 8.0–8.2.

Fig. 8 signifies that the content of Na-sol decreases as Na desorption occurs, which causes a higher pH of FW (by an alkaline reaction), so the lower the Na-sol, the higher the pH of the FW.

### 4.4 Relationship between soil pH and Na-sol

In stages 4 to 5, the decline in soil pHe was caused by lowering the water table during pond preparation for supplying of post-larvae, resulting in intense sulfurization. This is the overall process by which sulfuric acid and sulfate salts are produced from the oxidation of sulfides [5,6]. The low pH was accompanied by acidity (Eq. (2)) from the lower Cpr layer, which had an upward flux into the upper layers through capillary action [42,43].

In stages 1 to 4, the surS and subS of the LSA and HSA had varied pHe values around 5.0–6.0 (Fig. 9a–b). As indicated by Ref. [44] when the soil pH is less than 4, the bioavailability of essential nutrients is limited [45]. stated that most metal ions, particularly Al, are excessively mobilized in the soil; therefore, the soil pHe in LSAs and HSAs in Fig. 9a–b shows that their pH values are in the range of 5.0–6.0, which is considered to not restrict the growth of rice plants.

After stage 5, the pH of surS and subS increased back to its original value. According to Ref. [46], flooded seawater on acidified soils will significantly increase the pH of the soil [25]. found that in the sulfuric layer from the ASS, saline intrusion leads to a decrease in the amount of soil exch. Al^3+^ and a decrease in water-soluble Al as it is precipitated, thereby reducing the acidity of the soil. This indicates that saltwater flooding could be an effective strategy in remediating the acidity of ASS.

There was a model hypothesis about Na_2_SO_4_ that is generated in saline ASS, as [6] proposed that the Na-sol is considered to be present in the form of Na_2_SO_4_. From the deeper soil layer, the Na_2_SO_4_ salts could migrate to the topsoil, with S originating from the oxidation of pyrite and Na originating from the Na^+^ on the exchange position, which was replaced by ion H+, as this H^+^ was produced by pyrite oxidation and hydrolysis of Fe^3+^. In LSAs and HSAs, pond drying for stocking post-larvae is performed in January every year; this leads to the underground water table being deeper by 80 cm from the top soil, of which the sulfidic horizon is exposed to the air (Fig. 4). As a result of sulfurization, pyrite oxidation took place in stage 5, decreasing the pH of subS in LSAs and HSAs below 5.0 (Fig. 9a–b). Therefore, as in the subS of HSAs, the Na-sol of subS dropped from 65.0 to 51.0 mmol L^−1^ along with the pH decrease. Then, the transformation of exch. Na ^+^ into Na_2_SO_4_ salt took place and it rises to surS (Fig. 9b). In saline soil, usually, sulfate is less soluble than Cl and the sulfate salt concentration in seawater is usually much smaller. However, in areas influenced by oxidizing sulfide, sulfate can build-up in large amounts; therefore, many sulfate salts are predominantly found in ASS landscapes [27].

Gypsum is calcium sulfate (CaSO_4_). Usually, gypsum has water associated in its molecular structure (CaSO_4_·2H_2_O). Gypsum application is the most effective way to improve sodic soil to remove the exch. Na^+^. Ca^2+^ is exchanged with Na^+^, which is then leached out as a soluble salt such as Na_2_SO_4_. CaSO_4_ or CaCl_2_ also increases permeability by increasing the electrolyte concentration [56]. Using lime (CaCO_3_) is not recommended in sodic soil because it is not soluble at high pH levels, but CaSO_4_ is recommended for use in reclaiming sodic soils because it is calcium-rich and dissolves at high pH. Pyrite is also recommended for use in the reclamation of sodic soils because its oxidation produces sulfuric acid, which dissolves calcium carbonate and removes exch. Na^+^ from the solid phase [47]. In acidic conditions of saline acid sulfate soil, the dissolution of lime by sulfuric acid is a necessary condition to provide calcium for the improvement of sodic soil [29].

In Vietnam, lime is commonly used to improve acidic soils, but its effectiveness on saline soils has not been widely evaluated. Gypsum is the raw material of CaSO_4_, and it is considered an effective material in improving saline soil; however, it is mainly imported [48] and has a higher price, so it is not commonly used in agriculture.

There has been some evidence that the use of CaCO_3_ effectively improves saline ASS and rice yields in the MD. During the 2016 rainy season, field trials on rice were carried out on saline ASS in Long My, Bac Lieu, in the coastal area of the MD. The use of 1.8 Mg CaCO_3_ ha^−1^ led to an increase in exch. Ca^2+^ and reduced exch. Na ^+^ adsorbed in the soil layer, 0–20 cm. This soil improvement resulted in a higher grain yield through increased panicle number per m^2^ and grain number per panicle [49]. Another rice experiment conducted on saline ASS in Kien Giang, with the application of 0.9 Mg CaCO_3_ ha^−1^, led to reduced exch. Na^+^ and increased exch. Ca^2+^ and rice yield [50].

### 4.5 Dynamics of soil exch. Cations in HSA soils

Stage 1 is the beginning of the rainy season; the exch. base cations (Na^+^, Mg^2+^, Ca^2+^, and K^+^) were still high because of the remaining salts introduced by seawater during shrimp farming (stage 6). The exch. base cations decreased steadily from stages 1 to 2 and 3; the reduction occurred simultaneously with the dissolved salts in the FW being continuously discharged in the rainy season.

Even though there were variations in every base cation of surS and subS in six stages, the proportions of cations were always Na > Mg > Ca > K (Fig. 10a and b). In seawater, more than 99% of the salinity included six ions, which are in order of decreasing abundance: chloride (Cl^−^), sodium (Na^+^), sulfate (SO_4_^2−^), magnesium (Mg^2+^), calcium (Ca^2+^), and potassium (K^+^). The proportions of these ions in seawater remain the same everywhere [51]. At equivalent solution concentrations, the amounts of Ca^2+^ and Mg^2+^ are more strongly adsorbed by the exchange complex than Na^+^; however, with a higher concentration of sodium cations in HSA soil, Na^+^ was the predominantly adsorbed cation [19].

There was an increase in exch. base cations in stage 4, which coincides with the time of saltwater intrusion, leading to Na^+^ in surS in stages 4, 5, and 6 increasing continuously, with values of 6.3, 7.0, and 9.2 mmol kg^−1^, respectively (Fig. 10a). However, the Na^+^ in subS in stages 4, 5, and 6 had values of 5.7, 5.8, and 6.9 mmol kg^−1^, respectively, barely increasing in stage 5 as it was involved in sulfurization, leading to reduced adsorbed Na^+^ in subS (Fig. 10b).

K^+^ in surS and subS showed little variation over the six stages and had an average value of 1.0 mmol kg^−1^, which was 4–5 times lower than exch. Mg^2+^ and Ca^2+^, respectively. Exch. Mg^2+^ had an average value of 4–6 mmol kg^−1^ over the six stages. The value of exch. Mg^2+^ increased in stage 4, and stabilized in stages 5 and 6. Exch. Ca^2+^ had a mean value that was lower than that of exch. Mg^2+^ by about 0.5 units, and its variation was almost parallel to exch. Mg^2+^, except at stage 6, when exch. Ca^2+^ reached as high as exch. Mg^2+^. This increase in Ca^2+^ was a result of lime application during the farmers’ shrimp pond sanitation (Fig. 3). The increase in acidity by hydrolysis of H^+^ and Al^3+^ from the ASS [52,53] results in higher acidity of the solution and enhances the dissolution of CaCO_3_ and MgCO_3_. When the pH drops from around 8 to around 6, the solubility of CaCO_3_ in water may increase 20-fold [54].

Equation (4) indicates that the higher the acidity (lower pH), the more Na-sol is generated from exch. Na^+^. transformation; the result is that the exch. Na^+^ decreased. As indicated by Ref. [55], soil with high exch. Na^+^ content will have high Na-sol.

### 4.6 Dynamics of BS and ESP in HSA soils

The BS of surS dropped quickly from the beginning of the rainy season because salts were effectively leached out from the rice-shrimp fields (Fig. 11a). The BS of subS in stages 2 to 3 were below 50% because these BS related with soil pHe, which were around 5.0 (Fig. 9b).

In this RSF, the surS and subS of HSAs during rice cultivation had the ESP greater than 15% (Fig. 11b), EC_e_ greater than 0.4 S m^−1^ (Fig. 6b), and pHe less than 8.0 (Fig. 9b); therefore, this HSA soil was classified as saline–sodic soil [56]. Moreover, the LSA had the ESP lesser than 15% (Tabale 4), EC_e_ greater than 0.4 S m^−1^ (Fig. 6b), and pHe less than 8.0 (Fig. 9a); therefore, this LSA soil was classified as saline soil. Unlike sodic soils, as long as soluble salts are present, exch. Na^+^ is not a problem because the flocculation and water permeability of the soil is still good [19]. Therefore, in the reclamation of saline–sodic soils, the excess soluble salts must be leached downward, while exch. Na ^+^ needs to be replaced by Ca^2+^ and Mg^2+^; otherwise, the soil will become sodic [57].

## 5 Conclusion

The soil pH of both soils in the six stages were in the range of 5.0–6.0, with the exception of stage 5, where the pH dropped to 4.5. During the rice growth stage, the ECe of HSA soil was 0.7–0.8 S m^−1^, which was more than twice that of LSA soil. However, the ECe of FW in the two soils was about 0.2 S m^−1^; under these conditions, rice cultivation in saline soils after shrimp culture showed little damage to rice plants.

In the six stages of RSF operation, three main chemical processes occur: Na^+^ hydrolysis, sulfurization, and Na^+^ transformation; they occur during desalination for rice cultivation and during salination for shrimp farming. These processes were identified by large variations in chemical indicators and by regression analysis. Chemical indicators include pH of FW, pHe of soil, and concentration of Na-sol, which is especially seen more clearly for HSAs. The *Na* ^*+*^ *hydrolysis* that occurred in the desalinating phase, when excess soluble salts were leached by rainwater while exch. Na^+^ in the soil still remained, led to a spectacular increase in the pH of FW in stage 2. *Sulfurization* emerges during the salinization phase, as a consequence of the pond drying stage 5. This process produces acidity, which leads to exch. Na^+^ being displaced by H^+^ ions, which then become Na-sol and can migrate to the topsoil (*Na* ^*+*^ *transformation*). Multivariate regression analysis indicated that soil exch. Na^+^ was positively correlated with pH and Na-sol. Therefore, by producing acidity, sulfurization is responsible for reducing exch. Na^+^ absorption in the soil. Thus, these three processes eventually lead to the desorption of Na^+^.

As the “saline acid sulfate soil” in the coastal MD is formed from alluvial materials, especially the “typic Sulfaquepts” soil, when under the influence of salinity, the chemical properties of the soil are quite similar. Therefore, whether the soil is from the east or west of the coastal MD, the research results obtained are applicable.

Furthermore, under the condition that sulfurization occurs in saline ASS, the use of CaCO_3_ instead of CaSO_4_ has been shown to limit the amount of Na ^+^ absorbed in the soil. Therefore, chemical processes are simultaneously promoted from active soil preparation and liming for rice and shrimp farming, which helped to reduce the long-term build-up of salinity, Na^+^ adsorption, and acidity that adversely affect RSF production.

## Funding statement

This work was carried out with financial support from the Vietnamese Government under “The Scientific and Technological Program to support the National Targeted Program on Coping with Climate Change”, Code KHCN-BDKH-57.

## Author contribution statement

Conceived and designed the experiments – Ngo Ngoc Hung; Ngo Phương Ngoc.

Performed the experiments – Nguyen Van Qui; Le Van Dang; Ngo Phuong Ngoc.

Analyzed and interpreted the data – Ngo Ngoc Hung; Ngo Phuong Ngoc; Le Van Dang.

Contributed reagents, materials, analysis tools or data – Nguyen Van Qui; Ngo Phuong Ngoc.

Wrote the paper – Ngo Phuong Ngoc; Ngo Ngoc Hung.

## Additional information

No additional information is available for this paper.

## Declaration of competing interest

The authors declare no conflict of interest.

## Figures and Tables

**Table S1:** Time of salinity and Salinity (‰) of agro-ecological zone of Bac Lieu province.

Ecological zone		Duration of saline water intrusion	Salinity (‰)
Brackish water	Low salinity	From 3 to 6 months	From 4 to 6
	High salinity	From 3 to 6 months	More than 10
	Long-duration high salinity	More than 6 months	More than 10
Salt water		Year-round	More than 10

**Table S2:** Dataset on soil properties of acid sulfate soil (surS and subS) in low salinity area (HD1-HD10) and high salinity area (PL1-PL10), Bac Lieu province.

No	Soil layer	Salinity area	Code	Particle size distribution (%)			K_sat_ (mm h^−1^)	O·C (%)	CEC (cmolc^+^kg^−1^)
				Sand (0.05–2.00 mm)	Silt (0.002–0.05 mm)	Clay (<0.002 mm)			
1	surS	LSA	HD1	2	38	60	31.5	3.79	20.9
2	surS	LSA	HD2	2	46	52	36.8	1.68	18.5
3	surS	LSA	HD3	3	40	57	34.2	2.02	19.1
4	surS	LSA	HD4	1	43	56	43.5	2.22	19.0
5	surS	LSA	HD5	3	40	57	27.3	2.79	22.7
6	surS	LSA	HD6	3	48	49	44.6	2.96	18.4
7	surS	LSA	HD7	2	44	54	37.8	2.27	18.5
8	surS	LSA	HD8	3	47	50	53.5	2.34	20.0
9	surS	LSA	HD9	2	55	43	60.6	1.75	13.2
10	surS	LSA	HD10	2	50	48	56.2	2.03	17.1
11	subS	LSA	HD1	3	47	50	35.4	2.39	14.1
12	subS	LSA	HD2	3	38	59	17.8	2.52	18.6
13	subS	LSA	HD3	2	38	60	14.4	2.15	19.8
14	subS	LSA	HD4	4	37	59	17.5	2.64	19.7
15	subS	LSA	HD5	3	39	58	13.1	2.53	17.1
16	subS	LSA	HD6	4	36	60	12.1	2.56	19.8
17	subS	LSA	HD7	4	40	56	8.0	1.75	21.6
18	subS	LSA	HD8	3	36	61	14.4	2.21	20.8
19	subS	LSA	HD9	4	37	59	19.0	2.29	18.5
20	subS	LSA	HD10	2	35	63	10.0	2.44	21.2
21	surS	HSA	PL1	7	30	63	24.4	2.92	24.1
22	surS	HSA	PL2	6	49	45	74.5	2.32	19.3
23	surS	HSA	PL3	10	45	45	48.6	1.84	20.7
24	surS	HSA	PL4	4	31	65	23.1	3.59	24.1
25	surS	HSA	PL5	9	30	61	32.4	2.96	23.5
26	surS	HSA	PL6	5	36	59	28.2	3.02	23.0
27	surS	HSA	PL7	9	52	39	75.9	1.63	24.2
28	surS	HSA	PL8	8	31	61	24.8	3.24	23.4
29	surS	HSA	PL9	11	34	55	44.7	1.83	23.2
30	surS	HSA	PL10	7	37	56	35.9	2.36	22.2
31	subS	HSA	PL1	3	42	55	12.3	1.78	21.4
32	subS	HSA	PL2	3	43	54	21.5	3.03	18.4
33	subS	HSA	PL3	9	28	63	7.8	2.83	22.4
34	subS	HSA	PL4	7	35	58	13.7	2.43	20.3
35	subS	HSA	PL5	3	36	61	15.3	2.39	21.7
36	subS	HSA	PL6	9	26	65	4.2	2.96	24.4
37	subS	HSA	PL7	9	29	62	15.0	2.66	24.2
38	subS	HSA	PL8	8	33	59	25.3	2.21	23.8
39	subS	HSA	PL9	7	37	56	21.7	1.94	23.9
40	subS	HSA	PL10	7	33	60	15.8	1.91	18.5
