# Supplementary material for: Chemical processes and sustainability of rice-shrimp farming on saline acid sulfate soils in mekong delta
Source: Heliyon. 2023 Feb 6;9(2):e13532. doi: 10.1016/j.heliyon.2023.e13532 (PMC9929475; doi:10.1016/j.heliyon.2023.e13532)
Supplement: Multimedia component 1 [file mmc1.docx]

*Supplementary Material*

Table S1. Time of salinity and Salinity (‰) of agro-ecological zone of Bac Lieu province.

| **Ecological zone** | | **Duration of saline water intrusion** | **Salinity (**‰**)** |
| --- | --- | --- | --- |
| Brackish water | Low salinity | From 3 to 6 months | From 4 to 6 |
|  | High salinity | From 3 to 6 months | More than 10 |
|  | Long-duration high salinity | More than 6 months | More than 10 |
| Salt water | | Year-round | More than 10 |

Table S2. Dataset on soil properties of acid sulfate soil (surS and subS) in low salinity area (HD1-HD10) and high salinity area (PL1-PL10), Bac Lieu province.

| **No** | **Soil layer** | **Salinity area** | **Code** | **Particle size distribution (%)** | | | **K_sat_ (mm h^−1^)** | **O.C (%)** | **CEC (cmolc^+^kg^-1^)** |
| --- | --- | --- | --- | --- | --- | --- | --- | --- | --- |
|  |  |  |  | **Sand (0.05–2.00 mm)** | **Silt (0.002–0.05 mm)** | **Clay (<0.002 mm)** |  |  |  |
| 1 | surS | LSA | HD1 | 2 | 38 | 60 | 31.5 | 3.79 | 20.9 |
| 2 | surS | LSA | HD2 | 2 | 46 | 52 | 36.8 | 1.68 | 18.5 |
| 3 | surS | LSA | HD3 | 3 | 40 | 57 | 34.2 | 2.02 | 19.1 |
| 4 | surS | LSA | HD4 | 1 | 43 | 56 | 43.5 | 2.22 | 19.0 |
| 5 | surS | LSA | HD5 | 3 | 40 | 57 | 27.3 | 2.79 | 22.7 |
| 6 | surS | LSA | HD6 | 3 | 48 | 49 | 44.6 | 2.96 | 18.4 |
| 7 | surS | LSA | HD7 | 2 | 44 | 54 | 37.8 | 2.27 | 18.5 |
| 8 | surS | LSA | HD8 | 3 | 47 | 50 | 53.5 | 2.34 | 20.0 |
| 9 | surS | LSA | HD9 | 2 | 55 | 43 | 60.6 | 1.75 | 13.2 |
| 10 | surS | LSA | HD10 | 2 | 50 | 48 | 56.2 | 2.03 | 17.1 |
| 11 | subS | LSA | HD1 | 3 | 47 | 50 | 35.4 | 2.39 | 14.1 |
| 12 | subS | LSA | HD2 | 3 | 38 | 59 | 17.8 | 2.52 | 18.6 |
| 13 | subS | LSA | HD3 | 2 | 38 | 60 | 14.4 | 2.15 | 19.8 |
| 14 | subS | LSA | HD4 | 4 | 37 | 59 | 17.5 | 2.64 | 19.7 |
| 15 | subS | LSA | HD5 | 3 | 39 | 58 | 13.1 | 2.53 | 17.1 |
| 16 | subS | LSA | HD6 | 4 | 36 | 60 | 12.1 | 2.56 | 19.8 |
| 17 | subS | LSA | HD7 | 4 | 40 | 56 | 8.0 | 1.75 | 21.6 |
| 18 | subS | LSA | HD8 | 3 | 36 | 61 | 14.4 | 2.21 | 20.8 |
| 19 | subS | LSA | HD9 | 4 | 37 | 59 | 19.0 | 2.29 | 18.5 |
| 20 | subS | LSA | HD10 | 2 | 35 | 63 | 10.0 | 2.44 | 21.2 |
| 21 | surS | HSA | PL1 | 7 | 30 | 63 | 24.4 | 2.92 | 24.1 |
| 22 | surS | HSA | PL2 | 6 | 49 | 45 | 74.5 | 2.32 | 19.3 |
| 23 | surS | HSA | PL3 | 10 | 45 | 45 | 48.6 | 1.84 | 20.7 |
| 24 | surS | HSA | PL4 | 4 | 31 | 65 | 23.1 | 3.59 | 24.1 |
| 25 | surS | HSA | PL5 | 9 | 30 | 61 | 32.4 | 2.96 | 23.5 |
| 26 | surS | HSA | PL6 | 5 | 36 | 59 | 28.2 | 3.02 | 23.0 |
| 27 | surS | HSA | PL7 | 9 | 52 | 39 | 75.9 | 1.63 | 24.2 |
| 28 | surS | HSA | PL8 | 8 | 31 | 61 | 24.8 | 3.24 | 23.4 |
| 29 | surS | HSA | PL9 | 11 | 34 | 55 | 44.7 | 1.83 | 23.2 |
| 30 | surS | HSA | PL10 | 7 | 37 | 56 | 35.9 | 2.36 | 22.2 |
| 31 | subS | HSA | PL1 | 3 | 42 | 55 | 12.3 | 1.78 | 21.4 |
| 32 | subS | HSA | PL2 | 3 | 43 | 54 | 21.5 | 3.03 | 18.4 |
| 33 | subS | HSA | PL3 | 9 | 28 | 63 | 7.8 | 2.83 | 22.4 |
| 34 | subS | HSA | PL4 | 7 | 35 | 58 | 13.7 | 2.43 | 20.3 |
| 35 | subS | HSA | PL5 | 3 | 36 | 61 | 15.3 | 2.39 | 21.7 |
| 36 | subS | HSA | PL6 | 9 | 26 | 65 | 4.2 | 2.96 | 24.4 |
| 37 | subS | HSA | PL7 | 9 | 29 | 62 | 15.0 | 2.66 | 24.2 |
| 38 | subS | HSA | PL8 | 8 | 33 | 59 | 25.3 | 2.21 | 23.8 |
| 39 | subS | HSA | PL9 | 7 | 37 | 56 | 21.7 | 1.94 | 23.9 |
| 40 | subS | HSA | PL10 | 7 | 33 | 60 | 15.8 | 1.91 | 18.5 |
